# Rapid Biosynthesis of Silver Nanoparticles Using Pepino (*Solanum muricatum*) Leaf Extract and Their Cytotoxicity on HeLa Cells

**DOI:** 10.3390/ma9050325

**Published:** 2016-04-28

**Authors:** Mónica Gorbe, Ravishankar Bhat, Elena Aznar, Félix Sancenón, M. Dolores Marcos, F. Javier Herraiz, Jaime Prohens, Abbaraju Venkataraman, Ramón Martínez-Máñez

**Affiliations:** 1 Instituto Interuniversitario de Investigación de Reconocimiento Molecular y Desarrollo Tecnológico (IDM), Unidad Mixta Universitat Politècnica de València-Universitat de València, Camino de Vera s/n, Valencia 46022, Spain; mogormo@upvnet.upv.es (M.G.); elazgi@upvnet.upv.es (E.A.); fsanceno@upvnet.upv.es (F.S.); mmarcos@qim.upv.es (M.D.M.); 2 CIBER de Bioingeniería, Biomateriales y Nanomedicina (CIBER-BBN), Valencia 46022, Spain; 3 Materials Chemistry Laboratory, Department of Chemistry, Gulbarga University, Gulbarga, Karnataka 585106, India; kujalliravi@gmail.com (R.B.); raman.dms@gmail.com (A.V.); 4 Biological Research Innovation Centre and Solutions LLP, Bengaluru, Karnataka 56004, India; 5 Departamento de Química, Universitat Politècnica de València, Camino de Vera s/n, Valencia 46022, Spain; 6 Instituto de Conservación y Mejora de la Agrodiversidad Valenciana, Universitat Politècnica de València, Camino de Vera 14, Valencia 46022, Spain; fraherga@upvnet.upv.es (F.J.H.); jprohens@btc.upv.es (J.P.)

**Keywords:** biosynthesis, silver nanoparticles, *Solanum muricatum*, microwave irradiation, cytotoxic studies

## Abstract

Within nanotechnology, gold and silver nanostructures have unique physical, chemical, and electronic properties [1,2], which make them suitable for a number of applications. Moreover, biosynthetic methods are considered to be a safer alternative to conventional physicochemical procedures for both the environmental and biomedical applications, due to their eco-friendly nature and the avoidance of toxic chemicals in the synthesis. For this reason, employing bio routes in the synthesis of functionalized silver nanoparticles (FAgNP) have gained importance recently in this field. In the present study, we report the rapid synthesis of FAgNP through the extract of pepino (*Solanum muricatum*) leaves and employing microwave oven irradiation. The core-shell globular morphology and characterization of the different shaped and sized FAgNP, with a core of 20–50 nm of diameter is established using the UV-Visible spectroscopy (UV-vis), field emission scanning electron microscopy (FESEM), transmission electron microscopy (TEM) and Zeta potential and dynamic light scanning (DLS) studies. Moreover, cytotoxic studies employing HeLa (human cervix carcinoma) cells were undertaken to understand FAgNP interactions with cells. HeLa cells showed significant dose dependent antiproliferative activity in the presence of FAgNP at relatively low concentrations. The calculated IC_50_ value was 37.5 µg/mL, similar to others obtained for FAgNPs against HeLa cells.

## 1. Introduction

Metal nanoparticles (MNP) present unique physical (e.g., plasmonic resonance and fluorescent enhancement), chemical (e.g., catalytic activity enhancement), electronic, and antibacterial properties which make them appropriate for their application in fields including biosensing [3], photonics [4], electronics [5], antimicrobials [6], and in the biomedical field for drug delivery and targeting [7]. Moreover, functionalization of nanoparticles through the conjugation of certain chemicals is known to allow specific recognition of certain molecules and biomolecules, which may enhance efficacy of nanoparticles in specific applications and reduce side health and environment effects. Capping moieties are usually organic functional groups that coat the metallic core in a shell-like manner, so these nanoparticles are usually known as functional metal nanoparticles (FMNPs). Developing a reliable experimental protocol for the synthesis of FMNPs is a challenging issue in current nanotechnology research, particularly in the context of the recent drive to promote green technologies for their synthesis [8]. In this field, drawbacks associated with chemico-physical methods for the synthesis of AgNPs including the use of toxic chemicals, high temperature, pressure, and yielding of hazardous by-products, make it necessary to search for safer alternative methods. The ever growing need to develop a clean, nontoxic, and environmentally safe production processes for nanoparticles to reduce environmental impact, minimize waste, and increase energy efficiency has become essential in this field [9].

Recognizing the importance of developing eco-friendly methods for the synthesis of biologically active nanoparticles, scientists have additionally started looking into research relating to the synthesis of metallic nanoparticles embedded within bio moieties [10], possessing a core-shell morphology. The core being the metal nanoparticle (MNP), while the shell comprises of biochemical-moieties either from a plant extract [9,11,12,13,14,15,16,17], or from a microorganism [18,19,20], which can chemically interact with bio-organic molecules in a cellular environment. In both cases, MNP formation occurs when metal ions are reduced to its zero valent state. The reduction, employing microorganisms, is achieved through the reductase enzyme generated by the microorganism either by extracellular or intercellular route [21], while in case of plant extract the reduction process is through organic reducing agents present in the extract [9]. The mechanism of reduction employing plant extract is complicated as there may be 25–30 individual reducing components acting individually or combined in groups that lead to obtaining MNP. A detailed mechanism to understand the formation of MNPs using plant extracts is still unknown. However, highly reproducible and chemically homogeneous stable dispersions of MNPs are achieved [22,23,24,25]. The process to coat nanoparticles with bio-moieties acting as bio-conjugates has a crucial role in certain applications. In most cases, this biological shell presents the inherent ability to bind to biomolecules, allowing interaction with cells and offering the possibility of control cell responses [13,26,27,28,29]. From the structural point of view, shell consists of predominantly flavonoids [29,30].

Time consumption for synthesis of MNPs is the limitation of this process similar to the one employing microorganisms. However, some reports on the modified use of plant extracts have given insight into the possible organic moieties responsible for the metal ion reduction process along with greatly reducing the time duration for the synthesis [30,31,32,33]. The core-shell is nevertheless maintained. One of the trusted and successfully applied modified approaches consists in the use of a conventional microwave oven [11,14,18,34,35]. The extract on exposure to microwave radiation offers a rapid and uniform heating of the reaction medium and thus provides uniform nucleation and growth conditions for the synthesis of functionalized nanoparticles. Interestingly, capped MNPs (forming core-shell morphology) are obtained through this microwave assisted route, making the MNPs decidedly stable. The capping moieties from the extract and the metallic core form the core-shell morphology obtained. A judicious choice of the extract may confer to the FMNPs some properties of the functional groups used as shell, for example, to bind certain groups (organic/bio/cell/inorganic) for a desired application.

Extracts of other plants have also been studied by us [13,15,20,36] and other researchers [34,35,36,37,38]. Recently, MNPs have gained special attention for their promising potential as anticancer agent [12,13,31,36,39,40,41,42]. Characteristics as their intrinsic cytotoxicity, their larger surface area, and area:volume ratio, their easily tunable surface that allow conjugation or encapsulation with biological targeting or therapeutic molecules, and their optical characteristics, make them optimal materials for cancer theranostics [39,43] which try to combine diagnosis and therapy into a single agent. In earlier reports, some of us have used guava leaf extract to obtain functionalized gold and silver nanoparticles [34,35]. Guava leaf is known to possess anti-malignant character [44,45,46]. In this work, cell interaction studies showed that irregularly shaped Au nanoparticles were great anti-proliferative agents when compared with spherically shaped Au ones [34].

In this work, we focus in the formation and use of silver nanoparticles (AgNPs). Silver is known for its broad-spectrum antimicrobial activity. Moreover, several studies have reported the potential use of AgNPs as anticancer agents [39,42]. In this context, one of the challenging issues in current nanotechnology research is the development of a reliable experimental protocol for the synthesis of AgNPs with well-controlled morphological and physicochemical features for biomedical applications [8].

Thus, in the present work, we report the microwave assisted methodology for synthesizing functionalized silver nanoparticles, using the leaf extract of *Solanum muricatum* (*S. muricatum*). This species is usually referred to as “pepino”, and is an evergreen shrub native to the Andean regions, from southern Colombia to Bolivia and the coast of Peru [47]. It is mainly consumed as fruit around the world, but in some places it is also used as a vegetable. The fruit is very common in South America, although it is also cultivated in other countries like Spain, New Zealand, China, or USA [48]. Apart from nutritional aspects, the pepino plant is known for its anti-tumoral, antioxidative, antidiabetic, and anti-inflammatory properties [49,50,51]. Previous work from Ren *et al.* [50], found that an aqueous extract from *S. muricatum* was cytotoxic against tumor cell lines of prostate, liver, breast, and stomach triggering apoptosis signals. Although the molecular mechanisms of this cytotoxicity remain unclear, this work implies that pepino is a potent medicinal food.

**Figure 1:**
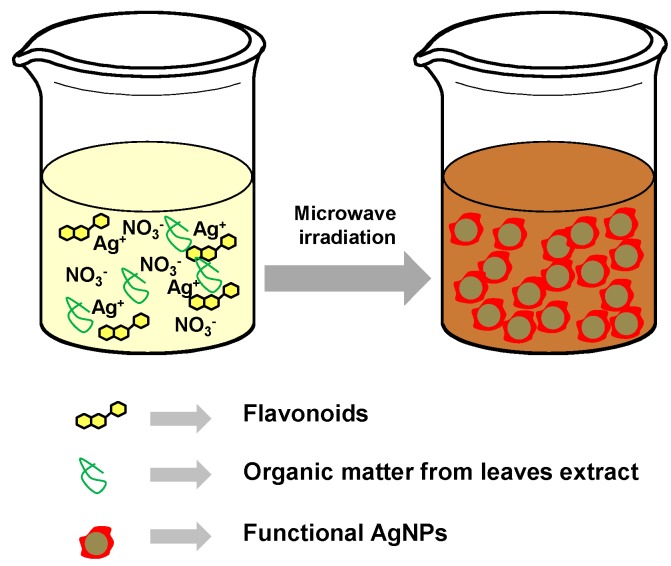
Pictorial representation of the microwave-assisted synthesis of AgNPs.

Present work shows the synthesis of FAgNP using pepino leaf extract as a reducing medium and microwave oven irradiation (see Figure 1). This paper also reports on the characterization of the FAgNPs employing different techniques. Formation of the FAgNPs is confirmed through the surface plasmon resonance (SPR) peak observed on the optical spectrum. The core-shell morphology of the FAgNP is established through optical images obtained by employing FESEM and TEM techniques and by dynamic light scattering measurements. Also, cytotoxic studies have been undertaken on HeLa (Human cervix carcinoma) cells. Cancer is considered one of the main causes of morbidity and mortality around the world, and the number of new cases increase every year. Moreover, cancer is not a single disease, it is a generic term for a large group of diseases that can affect any part of the body. For these reasons, cancer is one of the major public health concerns around the world [52]. Starting as localized focus of uncontrolled cell growth, cancer makes progress to a systemic disease which in many cases, and if the spread is not controlled, ends up with the death of the patient [53]. As we discussed above, nanotechnology, is an interdisciplinary field with great potential for its application in medicine and especially in cancer treatment and individualized therapy [54,55]. Nanoparticles with their small size are able to interact with larger biological molecules both outside and inside cells and they are internalized inside mammalian cells by uptake mechanisms as endocytosis. In this way, nanoparticles (NPs) offer a number of possibilities for cancer treatment and diagnosis [55,56].

## 2. Results

### 2.1. Synthesis and Characterization of Biofunctionalized Silver Nanoparticles (FAgNP)

In this work, the preparation of FAgNPs was achieved by treating with AgNO_3_ an extract of *S. muricatum* leaves obtained after the maceration by the immersion of chopped leaves in water and subsequent microwave irradiation of the mixture. In a typical synthesis, a reductant leaf extract was obtained from freshly collected leaves of pepino (*S. muricatum*) and exposed to microwaves using a conventional microwave oven at a frequency of 2.45 GHz for 180 s to denaturalize the enzymes and proteins present in the solution. In a following step, 40 mL of the extract were used to treat 800 mL of a 1 mM silver nitrate aqueous solution and exposed to microwave radiations in the same microwave oven for 90 s. When the mixture of the plant extract and the AgNO_3_ were irradiated, the colorless solution of the extract changed from pale yellow to intense reddish-brown color within 90 s, which evidenced the formation of FAgNP. This result is in agreement with previous studies indicating extracellular reduction process [13,14,15,19,20]. The yield of FAgNPs obtained by this microwave-assisted method using pepino leaf extract is comparable to chemical and physical methods, getting to complete the reduction process of the silver ions in 90 s. In the context of eco-friendly methods that use microorganisms and plants for the synthesis of FAgNPs, Balaji *et al.* [20] reported the extracellular biosynthesis of FAgNPs using the fungus *Cladosporium cladosporioides* in 78 h as Durán *et al.* [57] did in 72 h using biomass of *Fusarium oxysporum*. However, these methods are too slow for an industrial production of FAgNPs. More recently, other authors as Dubey *et al.* [58] managed to synthesize FAgNPs in 15 min using the extract of *Sorbus aucuparia* and Bhat *et al.* [15] reported the synthesis in some few hours using sun light irradiation and an extract of the mushroom *Pleurotus florida*. Compared with these reported data, our results indicated that microwave-assisted biosynthesis of FAgNPs is a procedure that allows reducing time needed for the reduction of silver ions and formation of nanoparticles [11,14,18,34,35].

**Figure 2:**
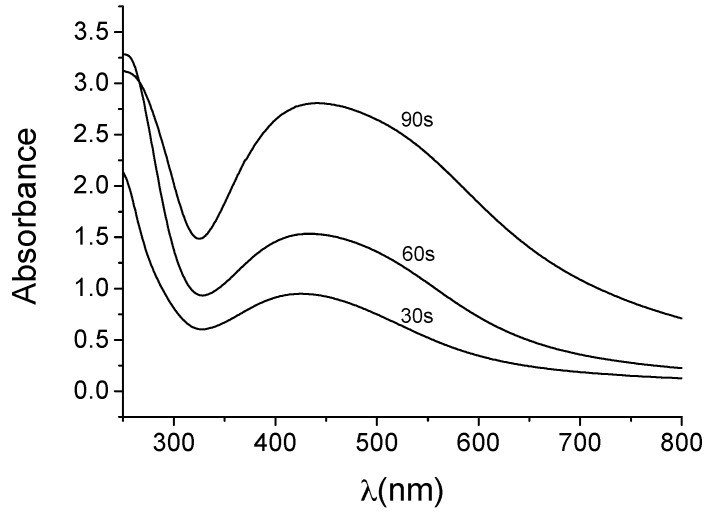
UV–visible spectra of biosynthesized FAgNPs. The surface Plasmon peak observed at 447 nm due to excitation of longitudinal plasmon vibrations confirms the presence of colloidal FAgNPs. Intensity of the peaks increases with exposure to microwave irradiation time. Peaks are broad and present no shoulders representative of the polidispersity of the sample and indicates a dipolar nature of the SPR.

The change in color was studied by UV-vis (JASCO Inc., Easton, MD, USA) to monitor the formation and stability of silver nanoparticles. UV-vis spectra were recorded at scheduled times and the results are shown in Figure 2. Changes in color of the solution take place within few seconds upon irradiation, and the characteristic surface plasmon resonance (SPR) signature of FAgNP was observed [19] as a band at 447 nm due to excitation of longitudinal plasmon vibrations [19]. Reports on the nature of the SPR peak and its characteristics have been discussed by several researchers, significantly by Taneja on the behavior of the deepening of the dip before the SPR peak and the effect of the dielectric medium on the shape index of the SPR peak [59]. The SPR peak in the present study is observed at 447 nm, which is consistent with reports from the literature in the study for the formation of FAgNPS employing different routes [19]. The SPR peak observed is broad and has no shoulders. The single broad peak without shoulders indicates polidispersity of the sample and a dipolar nature of the SPR. A complete formation of the FAgNPs is observed after 90 s of reaction. The formation of FAgNPs in a single step process is a significant feature when microwave irradiation is employed on plant extract in the preparation of MNPs [14].

It is interesting to note that, only the microwave irradiated extract of the *Solanum muricatum* leaves showed the formation of FAgNPs upon addition of AgNO_3_ solution, while the pre-irradiated extract did not show this behavior. It may thus be understood that the microwave irradiation of the aqueous extract contained useful organic moieties responsible for the chemical reduction of ionic Ag^+^ to Ag. In this context, the concept of electron transfer mechanisms for reduction of metal ions to form FAgNPs has been reported by several authors [30,37,38]. As the microwave irradiation inactivates enzymes and proteins, the reducing behavior of the extract is most likely due to the presence of flavonoids which are known to have the potential to act as reducing agents [32]. Flavonoids are phenolic compounds of the secondary metabolism of the plants. Sudha *et al.* [51] found high amounts of phenols and flavonoids on aqueous extracts of *S. muricatum*. The phytochemicals in *S. muricatum* displayed several bioactivities as antioxidative, antidiabetic, and antiinflammatory properties. Moreover the extracts also showed cytotoxic activity against cell lines of breast, stomach, ovarian, liver, lung, and prostate cancers [32,49,50,51] by triggering apoptosis.

**Figure 3:**
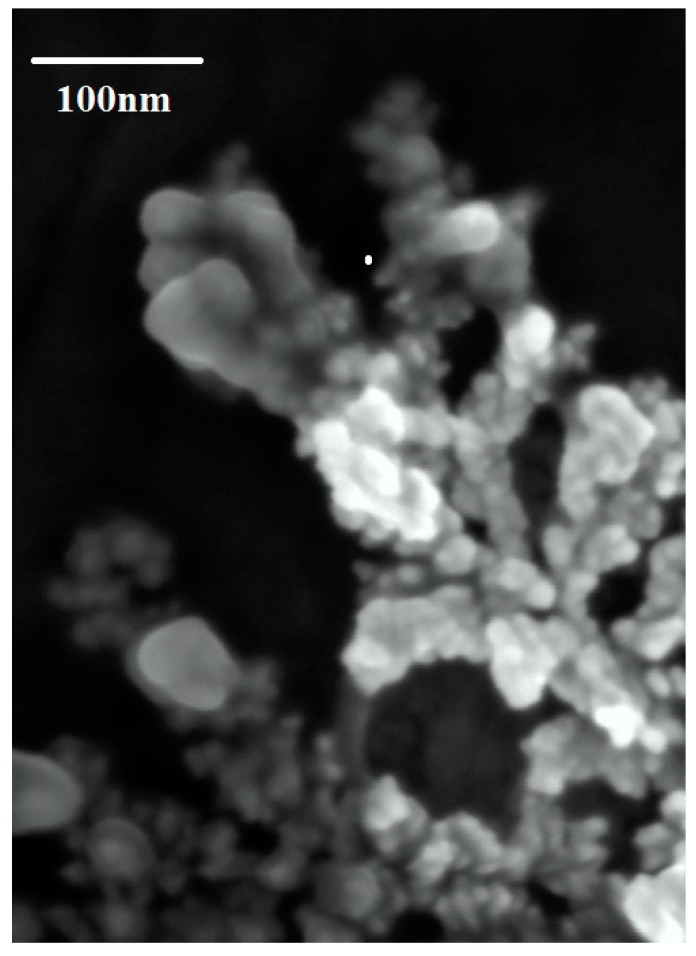
FESEM image of FAgNP using *Solanum muricatum* leaves extract. Figure shows FAgNP surrounded in a dense organic layer.

**Figure 4:**
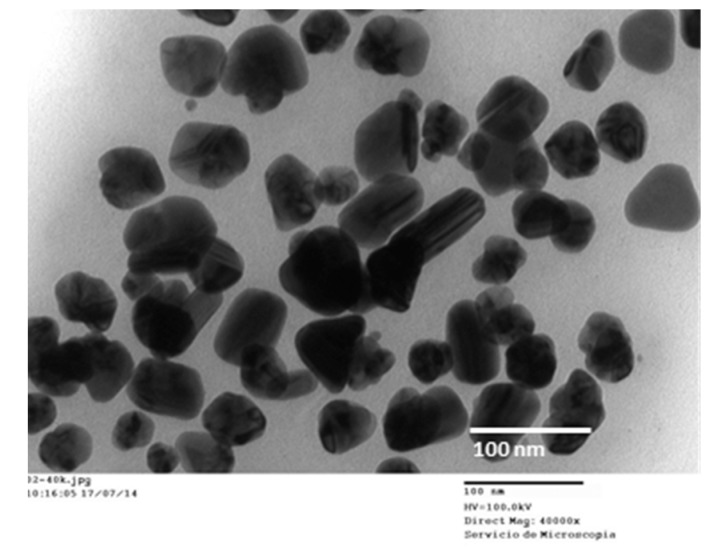
Transmission electron microscopy (TEM) images of representative FAgNPs. The obtained FAgNP are irregular in shape and its size is in the 20–80 nm range.

In a further step, the size and shape of the synthesized FAgNPs were studied by FESEM and TEM. FESEM samples were prepared by deposition of FAgNPs in powder form on a sticky conducting copper tape that was mounted on an aluminum disc. FESEM micrographs (Figure 3) show typical images of aggregated FAgNP possessing core-shell morphology of silver nanoparticles embedded in an organic shell with sizes below 100 nm. The particles are conjoined with their neighbors forming predominantly rounded core-shell morphology. The core size is around 30–50 nm and closer observation indicates irregular but mostly rounded shaped morphology. TEM studies provided further insight into the morphology and size of these nanoparticles. TEM samples were prepared by deposition of one drop of the reddish brown suspension on a carbon-coated copper TEM grid. Figure 4 shows representative TEM images of the obtained FAgNPs. Silver nanoparticles are irregularly shaped, well separated, and no agglomeration was observed. The size of the nanoparticles is in the 20–80 nm range with an average size of 59.34 ± 16.63 nm. A careful examination of the TEM images showed that the surface of silver nanoparticles was covered with an organic thin layer from the plant extract. However, if compared with FESEM images the shell seems to be partially ruptured or flared out, that may be due to the experimental conditions employed for TEM. In particular, TEM measurement uses ultra-high vacuum along with high voltage on a dried FAgNPs powder, which seems to be responsible for the partial decapping of the shell.

**Figure 5:**
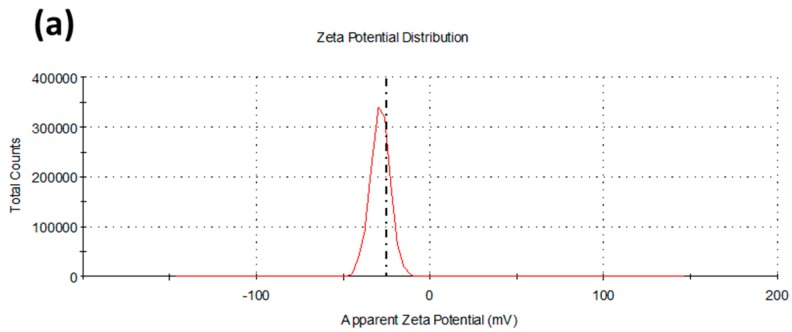

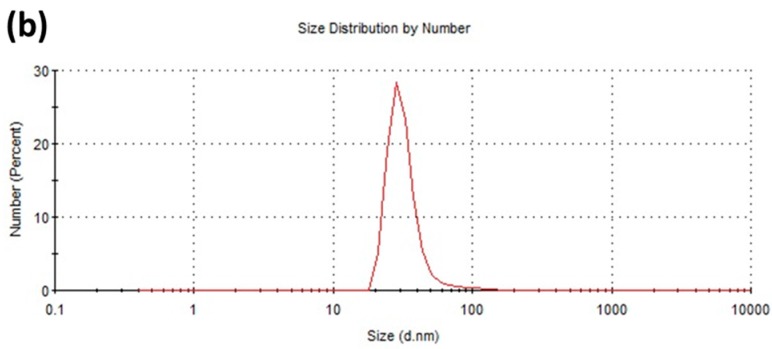
Dynamic light scattering (DLS) studies of the biosynthesized FAgNPs. (**a**) Zeta potential distribution; (**b**) Size distribution by number.

Dynamic light scattering measurements were performed to determine the hydrodynamic diameter of the prepared FAgNPs. The obtained size distribution is depicted in Figure 5a. A one-band size distribution in the 20–70 nm range was observed with a mean diameter of 40 nm. This unique peak also confirmed the absence of larger aggregates when FAgNPs were dispersed in aqueous media. Moreover, Zeta potential measurements were also carried out. A value of −28.75 ± 5.61 mV was obtained. Zeta potential is a crucial factor for the determination of the stability of suspensions of nanoparticles. This parameter is related with the surface charge of the nanoparticles. When particles in a colloidal system have large negative or positive Zeta potential, repulsion forces between them prevent aggregation. In contrast, when nanoparticles present low surface charges, the absence of repulsion forces favors aggregation. In this context, nanoparticle suspensions with Zeta potential values greater than +25 mV or lower than −25 mV usually form high stable suspensions. [60] The value of Zeta potential for our prepared FAgNPs indicates that the nanoparticles are highly stable. These results suggest that pepino leaf extract is not only a good bioreductant for the preparation of FAgNPs but also that the final organic shell prevents aggregation of nanoparticles.

### 2.2. Cytotoxicity Studies

Once prepared and fully characterized, we focused our attention to study the potential use of FAgNPs as an antiproliferative agent. With the increasing number of published articles about the use of different nanomaterials for the treatment of several diseases due to their cytotoxic properties, it has raised concerns about safety in medical use and *in vivo* effectiveness of these nanomaterials. The first report on the cytotoxic effect of FAgNPs synthesized from plant extracts was tested in MCF-7 breast cancer cells in 2013 [12]. Since then, several studies have evidenced that therapy with biosynthesized FAgNPs is a promising alternative for traditional anticancer treatments [39]. However, further investigations are needed, mainly related with safety issues associated to their use in humans and their effects on the environment [61].

In the present study, the inhibitory activity of pepino leaves extract and bio-synthesized FAgNPs was assessed for *in vitro* antiproliferative activity against HeLa human cervix cancer cells using the WST-1 method [62]. This colorimetric assay is based on the reactivity of mitochondria succinate-tetrazolium reductase system which is only active in metabolically intact cells. The enzymatic system cleaves the slightly red tetrazolium salt WST-1 to a soluble dark red formazan chromophore. Therefore, the amount of formazan dye formed directly correlates with the number of metabolically active cells in the culture. The change of color produced by the increased amount of formazan can be measured at 450 nm spectrophotometrically. Then, the obtained absorbance for each FAgNPs concentration, normalized using the absorbance of the untreated control wells, is represented. Data is adjusted to a sigmoidal curve and the inhibitory effect (IC_50_ value) is calculated as the concentration required of FAgNPs required inhibiting the growth of tumor cells in culture by 50% compared to untreated cells. The reduction of WST-1 can only occur in metabolically active cells; therefore, the change in color of the culture media is a measure of viability of cells. Absorbance values lower than control cells indicate a reduction in the viability of the cultures. In opposition, an increase in the absorbance value of the media is correlated with an increase in cells viability. The percentage of cell proliferation inhibition for each treatment is then calculated in relation to controls which were considered as 100% of cell proliferation.

**Figure 6:**
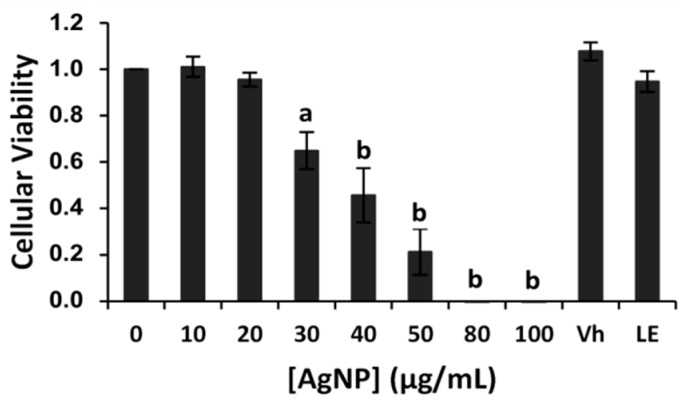
Cytotoxic effect of the biofunctionalized synthesized FAgNP against human cancer HeLa cells. Data are expressed as means ± standard error of the mean (SEM) for five independent experiments with six replicates each. Statistical significance of results was studied performing Student *t*-test with a *p* value (*p*). *p* > 0.05 as a minimal level of significance. Asterisk indicates statistically significant differences between untreated control and synthesized FAgNPs treated cells (a) *p* < 0.05 v/s corresponding controls (b) *p* < 0.01 v/s corresponding controls. Vh (Vehicle; distilled water); LE (leaves extract).

As it was expected (*vide infra*), when the effectiveness of synthesized FAgNPs against HeLa cells was tested, a dramatic decrease in cell viability was registered as the concentration of FAgNPs increased at dilutions ranging from 20 to 100 µg/mL (10, 20, 30, 40, 50, 80, 100 μg/mL, Figure 6). Clearly, a significant dose-dependent reduction in cell viability was observed. From the represented data, the half maximal inhibitory concentration (IC_50_) was calculated and an IC_50_ value of 37.5 µg/mL was obtained. No inhibitory effect in cell viability was observed in samples at dilutions lower than 20 µg/mL of FAgNPs. When cultures are treated with the aqueous plant extract of *S. muricatum*, leaves (LE) or with only vehicle (Vh), which in this case is a treatment with the same amount of only solvent of the FAgNPs (distilled water), cells remained alive.

A clear antiproliferative effect of FAgNP was observed for HeLa cells. A similar inhibitory cell proliferation capability was found in other recently published works using biosynthesized FAgNPs from other organic extracts and tested against a HeLa cervix cancer cell line. Palaniappan *et al.* [63] have reported the cytotoxic effect of FAgNP from an aqueous extract of *Cymodocea serrulata* on HeLa cells with an IC_50_ value of 34.5 μg/mL; Balakumaran *et al.* [64], noticed an IC_50_ value of 27.5 µg/mL for HeLa cells when using FAgNPs obtained from an extract of the endophytic fungus *Guignardia mangiferae*. In another study by Chanthini *et al.* [65] the medicinal sea grass *Cymodocea serrulata* was used to obtain FAgNPs, and the nanoparticles gave an IC_50_ value of 34.5 µg/mL also against HeLa cells. Also, Sreekanth *et al.* [66] obtained a dose dependent cytotoxicity of FAgNPs biosynthesized using *Nelumbo nucifera* with a minimum 56% and maximum of 83% of growth.

It is well documented that FAgNPs induce cell death through genotoxicity, loss of the cell membrane integrity, oxidative stress, and apoptosis [67]. Although the mechanisms of cytotoxicity induced by FAgNPs are poorly studied, several works evidence that it is mainly produced by oxidative stress [67,68,69]. Recent research has also shown that silver ions released by the silver nanoparticles play an important role in cellular death [70] and together show a synergistic effect [71]. Briefly, after the cellular uptake, particles are preferentially accumulated inside endosomes and lysosomes where acidic environment and high intracellular dissolved oxygen concentration produce reactive oxygen species (ROS). In this moment, the dissolution of silver nanoparticles to silver ions (Ag^+^) takes place through an oxidation reaction [42,72,73,74] and the partial inhibition of the cell ROS defense mechanisms [75]. In a further step, both FAgNPs and silver ions are released from lysosomes and are able to disrupt mitochondrial activity and further increase the production of intracellular ROS, which, simultaneously, would increase oxidation of FAgNPs and liberation of silver ions from them [73,74]. The sustained ROS production and silver ions release are responsible for cell structure damage. Moreover, FAgNPs and released Ag^+^ can interact with thiol groups in molecules present in the cytoplasm, cell membrane, and inner membrane of mitochondria, which might release lipid peroxide and increases permeation of the cell membrane and mitochondrial systems. Additionally, FAgNPs and Ag^+^ are also able to translocate and diffuse directly to the nucleus and trigger DNA abnormalities [68]. All of these referred mechanisms can trigger different cell death mechanisms as apoptosis and necrosis [76].

Several factors influence toxicity of FAgNPs such as dose, time, and size of the particles [12] but other factors such as shape, surface coatings, and charge and cell type might also be crucial in the FAgNPs toxicity. In particular, the toxicity of nanomaterials is related with their capability to react with organic moieties of biomolecules, which depends on the reactive surface area of the nanoparticles and therefore closely depends on the nanoparticle size [77]. Then, dose-dependent decrease in cell viability observed in our work can be explained by taking into account the increasing number of FAgNPs accumulated inside cells. In this situation, an increase in the amount of reactive surface area of nanoparticles is clear, which results in enhanced stress ultimately leading to cell death.

FAgNPs are toxic to the mammalian cells [78]. However, there is evidence that cytotoxic effects of FAgNPs are greater on cancer cells than on normal cell lines [65,79]. For instance, Ren *et al.* [50] showed that *S. muricatum* aqueous extracts are able to produce a selective cytotoxicity against cancer cell lines, three- to six-fold higher than that observed in normal cell lines. Here we have tested the cytotoxic capabilities of *S. muricatum* biosynthesized FAgNPs against HeLa cells. Although further experiments are needed to fully confirm the anticancer potential of these nanoparticles, it seems that both characteristics (*i.e.*, toxicity of AgNPs and *S.muricatum* extracts) can be acting in a synergistic manner and this could be a promising potential nanotherapeutic agent for cancer treatment.

## 3. Materials and Methods

### 3.1. General Remarks

Fresh leaves of pepino “El Camino” (*Solanum muricatum*) were picked directly from *S. muricatum* plants cultivated in the Institute for Conservation and Improvement of Valencian Agrodiversity (COMAV). Silver Nitrate (AgNO_3_) A.R grade was purchased from Sigma-Aldrich Química S.A. (Madrid, Spain). Solutions were prepared with double-distilled water. Microwave furnace, BLUEsky (Blue Sky Communications, Cheyenne, WY, USA) 2.45 GHz was used for heating of leaves extract to inactivate the enzymes present in the leaves and for the synthesis of FAgNP.

### 3.2. Green Synthesis of the Silver Nanoparticles

The process for extracellular maceration of *S. muricatum* has been followed as per our earlier work [35], along with some modification as discussed below:

In a typical synthesis, to obtain a reductant leaf extract, 5 g of freshly collected leaves of Pepino ‘El camino’ (*Solanum muricatum*) are washed with distilled water to remove any organic residue that may remain on the surface and perfectly dried. After that, the leaves are finely chopped in small pieces (approximately 1 cm × 1 cm) with a sterile scalpel and submerged in 100 mL of double distilled water in a 250 mL glass beaker. The humid leaf pieces of *S. muricatum* “El camino” were exposed to microwaves for 180 s to denaturalize the enzymes and proteins present in the solution in a conventional microwave oven at a frequency of 2.45 GHz. The raw extract obtained was collected by passing through Whatman filter paper 42 and the resultant filtrate is used for the reduction process of Ag^+^ to Ag. Then 40 mL of the extract were used to treat 800 mL of a 1 mM silver nitrate aqueous solution in a 1 L glass beaker and exposed to microwave radiation in the same microwave oven for 90 s. The changes in color of the solution take place within a few seconds, changing from pale green to reddish brown color evidences the formation of FAgNP.

FAgNPs are finally collected through ultracentrifugation at 9500 rpm for 20 min. The whole pellet obtained corresponds to the FAgNPs fraction, which was washed twice with double-distilled water and dried at 37 °C to obtain 52 mg of the final FAgNPs.

### 3.3. Characterization

The formation of FAgNP was verified by using JASCO V-650 spectrophotometer (JASCO Inc., Easton, MD, USA) operated at 1 nm resolution with optical length of 10 mm. Measurements of size and Zeta potential were studied by Dynamic Light Scattering (DLS) using a Zetasizer Nano ZS Analyzer (Malvern Instruments Ltd., Worcestershire, UK) in the range of 0.1–1000 µm. The structural morphology of FAgNP was also examined by JEOL TEM-1010 Electron microscope (JEOL USA, Inc., Peabody, MA, USA) working at 100 kV and by Field Emission Scanning Electron Microscopy FESEM using ZEISS Ultra 55 instrument (ZEISS, Oberkochen, Germany).

### 3.4. Cell Lines and Culture Conditions

HeLa human cervix adenocarcinoma cells were purchased from the German Resource Centre for Biological Materials (DSMZ). HeLa cells were grown under standard culture conditions (37 °C, 5% CO_2_) in DMEM medium (Sigma-Aldrich^®^, St. Louis, MO, USA) supplemented with 10% fetal bovine serum (FBS), and underwent passage twice a week in order to keep cells under appropriate growing conditions. For this, when cultures reach 90%–100% confluence, cells are detached from the bottom of the flask by trypsinization and diluted properly for each cell line.

### 3.5. Effects on Cell Growth/Viability

Both leaf extract (*S. muricatum*) and FAgNP solutions were evaluated for *in vitro* cytotoxicity on HeLa (Human cervix carcinoma) cells at different concentrations (10, 20, 30, 40, 50 80, 100 µg/mL) performing WST-1 (Cell proliferation reagent WST-1, Roche, Basel, Switzerland) assays.

The monolayer cell culture was washed with PBS and treated with 1.5 mL of trypsin-EDTA (Gibco^®^, Thermo Fisher Scientific Inc., Waltham, MA, USA), which breaks down proteins, until cells were detached from the bottom of the growing flask and well dispersed to obtain a homogeneous suspension of cultured cells. Then, cells were counted and 2.5 × 10^3^ cells per well were seeded in a 96-well microplate, adding 0.1 mL of diluted cell suspension in each well. After 24 h, the supernatant was removed and 100 µL of leaf extract or FAgNP water solution were added to the cells, depending on the treatment, and kept in incubation at 37 °C in 5% CO_2_ incubator for 72 h. Increasing concentrations used were 10, 20, 30, 40, 50, 80, 100 µg/mL in distilled water. Then, 7 µL of WST-1 were added to each well and further incubated 1 h until obtain the change of color in control cultures. Finally, absorbance of the cultures was measured at 450 nm using a microplate reader Thermo ScientificTM MultiskanTM FC Microplate Photometer (Thermo Fisher Scientific Inc., Waltham, MA, USA). Reference measurements were taken at 620 nm. Inhibitory effect (IC_50_) was calculated for the FAgNP concentration that is required to reduce absorbance of 50% of the control culture, based on the dose-response curve for different FAgNP concentrations as shown in Figure 6. Absorbance values that are lower than the control cell lines reveal a decline in the rate of cell proliferation. Conversely, a higher absorbance indicates an increase in the cell proliferation. Untreated culture wells of HeLa cells were considered as proliferative control. The percent inhibition of cell proliferation was calculated based on difference in inhibitory effect between treated cell lines and their respective controls, where 100% cell proliferation was taken from corresponding controls.

### 3.6. Statistics

All the results are expressed as mean ± standard error of the mean (SEM) of five independent experiments with six replicates of each condition. The difference in inhibitory effect at different doses between treated and corresponding controls was analyzed for statistical significance by performing Student *t*-test with *p* > 0.05 as a minimal level of significance. The IC_50_ value was calculated fitting toxicity data with a Sigmoidal Dynamic Curve 4 Parameter Fit using SigmaPlot version 13.0, from Systat Software, Inc., San Jose, CA, USA, www.sigmaplot.com, with a total number of fit iterations of 200.

## 4. Conclusions

To conclude, in the present investigation we successfully developed an environmental friendly synthesis method for the production of biosynthesized FAgNP by exploiting *S. muricatum* leaf extract as a potential bioreductant. In particular, FAgNPs were obtained by treating a solution AgNO_3_ with the extract of *S. muricatum* leaves and subsequent microwave irradiation of the mixture. The prepared nanoparticles have been characterized by different techniques such as UV-vis, FESEM, TEM, and DLS. The rapid biosynthetic method developed in this study for producing silver nanoparticles has distinct advantages over chemical methods such as a high biosafety, ecofriendliness, and nontoxicity to the environment. Finally, potential cytotoxicity studies of the nanoparticles against HeLa cells were conducted. From those experiments, an IC_50_ value of 37.5 µg/mL was estimated, which is in the range of other reported works where FAgNPs cytotoxicity against HeLa cells were tested.

## Abbreviations

The following abbreviations are used in this manuscript:

AgNP	Silver nanoparticles
DLS	Dynamic Light Scattering
FAgNP	Functionalized silver nanoparticles
FESEM	Field emission scanning electron microscopy
LE	Leaf extract
MNP	Metal Nanoparticles
ROS	Reactive oxygen species
SEM	Standard error of the mean
*S. muricatum*	*Solanum muricatum*
SPR	Surface plasmon resonance
TEM	Transmission electron microscopy
Vh	Vehicle
